# Space, time and complexity in plant dispersal ecology

**DOI:** 10.1186/s40462-014-0016-3

**Published:** 2014-08-01

**Authors:** Juan J Robledo-Arnuncio, Etienne K Klein, Helene C Muller-Landau, Luis Santamaría

**Affiliations:** Department of Forest Ecology & Genetics, INIA-CIFOR, Ctra. de la Coruña km 7.5, 28040 Madrid, Spain; INRA, UR546 Biostatistique et Processus Spatiaux (BioSP), Avignon, France; Smithsonian Tropical Research Institute, Apartado Postal 0843-03092 Panamá, Republica de Panamá; Spatial Ecology Group, Doñana Biological Station (EBD-CSIC), Sevilla, Spain

**Keywords:** Seed, Pollen, Gene flow, Dispersal kernel, Migration, Long-distance dispersal, Community ecology, Climate change

## Abstract

Dispersal of pollen and seeds are essential functions of plant species, with far-reaching demographic, ecological and evolutionary consequences. Interest in plant dispersal has increased with concerns about the persistence of populations and species under global change. We argue here that advances in plant dispersal ecology research will be determined by our ability to surmount challenges of spatiotemporal scales and heterogeneities and ecosystem complexity. Based on this framework, we propose a selected set of research questions, for which we suggest some specific objectives and methodological approaches. Reviewed topics include multiple vector contributions to plant dispersal, landscape-dependent dispersal patterns, long-distance dispersal events, spatiotemporal variation in dispersal, and the consequences of dispersal for plant communities, populations under climate change, and anthropogenic landscapes.

**Electronic supplementary material:**

The online version of this article (doi:10.1186/s40462-014-0016-3) contains supplementary material, which is available to authorized users.

## Introduction

Pollen and seed dispersal are essential functions of plants, with far-reaching consequences for reproduction, population and community dynamics, neutral and adaptive evolution and, ultimately, population and species persistence. Because an understanding of gene and individual movement capacities is critical to predicting the response of individuals, populations and species to ecosystem perturbation and climate change, the long-standing interest in plant dispersal has seen an upsurge in recent years. Extensive monographs have recently dealt with the ecology and evolution of dispersal of organisms in general [1] and of plants in particular [2]. Other more specific reviews (many of which are cited below) have focused on the mechanisms, consequences and measurement of passive and animal-mediated plant dispersal, considering different spatial and temporal scales and varied ecological, demographic and evolutionary settings.

**Figure 1:**
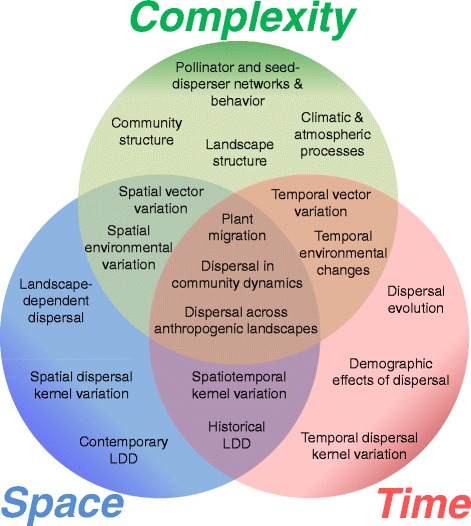
**Diagram of plant dispersal research topics considered in this study, each of which confronts challenges of spatial scale and heterogeneity, temporal scale and/or system complexity.**

Here, we pose eight general questions that we believe will define some of the research frontiers in plant movement ecology in the coming years. We do not attempt to answer these questions, or to exhaustively review the state-of-the-art in these areas, but rather offer our perspectives regarding a selection of important research topics, with an emphasis on specific empirical objectives and methods. The paper is oriented along three axes, representing three fundamental dimensions that challenge ecological inference and models in general, and dispersal ecology research in particular: space, time and complexity (Figure 1). Spatial scale and heterogeneity issues typically arise in long-distance dispersal (LDD) estimation and modeling, but also when characterizing dispersal variation among individuals, populations and regions, when assessing landscape effects on dispersal, or when measuring dispersal anisotropy. Temporal issues are inherent in studies examining dispersal fluctuations across years or dispersal seasons, and also arise when building up dispersal kernels from descriptions of instantaneous vector movement, when obtaining robust estimates of dispersal variation among individuals or populations, when inferring historical migration rates from genetic data, and when predicting long-term feedbacks between dispersal, demography and evolution. The dynamic complexity of environments, communities and ecosystems pervades most aspects of dispersal ecology research, from pollinator and seed-disperser networks, through the consequences of dispersal for population and community dynamics, to dispersal sensitivity to global change (Figure 1).

Within this broad framework, we first argue that mechanistically accounting for the relative contribution of multiple vectors to dispersal of particular plant species constitutes an essential basis for explaining and predicting dispersal patterns in spatiotemporally changing ecosystems (Section 1). Next, we examine how the interplay between vectors and environmental heterogeneity determine landscape-dependent seed and pollen deposition patterns that are missed by pure distance-dependent models (Section 2). We then focus on the broadest spatial scale by examining the measurement of long-distance dispersal across species’ ranges (Section 3). We continue by addressing the causes and consequences of variability in dispersal patterns among individuals and populations (Section 4), and over time (Section 5). Finally, we take a broader temporal perspective to consider the consequences of dispersal for plant communities (Section 6), populations under climate change (Section 7), and anthropogenic landscapes (Section 8).

## Review

### 1. What are the contributions of different vectors to plant dispersal?

It is now acknowledged that for many if not most plant species multiple vectors contribute to dispersal [3]: polychory (seed dispersal by multiple animal vectors) is widespread [4], ambophily (pollination by insects and wind) might be more common than previously thought [5], and mutualistic networks confirm the diversity of animal pollinators [6]. Knowing the variety of vectors for the species of interest is an essential initial step of dispersal studies, because different vectors may disperse propagules (defined here as pollen, seeds or spores) over contrasting spatial scales [7-9], their activity may fluctuate over different spatial and temporal scales, and they may respond differently to environmental and demographic changes. Of special interest is identifying vectors, vector characteristics, or environmental conditions responsible for LDD events, because they often result from nonstandard dispersal conditions [3,4,10-13] and contribute disproportionately to demography and population genetics (see Section 3). However, most intraspecific studies of seed and pollen dispersal have focused on a single vector.

The relative contribution of multiple vectors to dispersal can be investigated using empirical and modelling approaches. Dispersal kernels (i.e. the probability distribution of dispersal locations relative to the source location) can be estimated empirically based on direct observation of propagule deposition patterns at a sample of settling locations (Eulerian methods), or by tracking individual propagules (Lagrangian methods), most frequently over short to intermediate scales (e.g. [2,14-19]). Assessing the contribution of multiple vectors to dispersal based on these methods involves pairing each propagule deposition or transport event with the responsible vector, which may not be easy in practice. A few recent studies provide good examples of how to empirically investigate the contribution of multiple animal vectors to seed [20-23] and pollen [24] dispersal kernels, and we expect to see more such efforts in the future, including replication over different landscape configurations, environments and dispersal seasons. A complementary and more explanatory approach consists in modeling the dispersal kernel from mechanistic (process-based) considerations [25], and calibrating it using Eulerian [26] or Lagrangian [27] data. Mechanistic models provide excellent tools for evaluating the relative importance of different dispersal vectors, because they involve (i) inventorying the biological, ecological and environmental factors that impact propagule paths from emission to final deposition, and (ii) quantifying the probabilities associated with the different factors. For example, if wind speed, settling velocity and release height are fixed, the ballistic equation provides a unique dispersal location for a propagule passively dispersed by wind, assumed to follow a deterministic linear path. Integrating over probability distributions for wind speed, release height, propagule mass and area then results in a dispersal kernel [26,28]. Similarly, propagule dispersal by different animal vectors can be modeled with dispersal kernels that integrate observations of disperser movement and foraging behavior with models of seed retention [21] or pollen carry-over [29].

Mechanistic approaches are valuable for understanding landscape-dependent dispersal patterns (Section 2) and the occurrence of patchy or clumped dispersal resulting from correlated movement of propagules [30]. They are particularly useful for investigating LDD events and their associated vectors, because they can potentially inform accurate extrapolation to larger scales than those observed [25] (see Section 3). They can also assist in predicting the effects of spatiotemporal variation in the environment and in plant phenotypes on vector behavior and the distribution of dispersal distances (Sections 4 and 5). Future studies should further exploit mechanistic methods to (i) investigate the extent to which dispersal kernels are dynamic distributions subject to temporal and environmental influence, and (ii) identify the critical vectors and environmental variables with disproportionate impact on dispersal probabilities over short and long distances. For this purpose, it will be important to validate mechanistic predictions with alternative methods (e.g., direct observations or genetic approaches; see Section 3) over different spatial and temporal scales, and to cross-validate with independent data sets.

### 2. How can we better characterize landscape-dependent variation in seed and pollen deposition, and how can we better evaluate its consequences?

Historically, most studies of seed and pollen dispersal have described dispersal patterns exclusively in terms of the distribution of deposition distances from sources (e.g. [15,31]). Clearly, distance from sources is important in explaining variation in propagule deposition, and dispersal distance is also critical in determining the consequences of dispersal [32,33]. However, distance generally explains only a small fraction of variation in seed [34] or pollen [35] densities. The unexplained variation is important for post-dispersal success of individual seeds or pollen grains, and for population and community processes and patterns [36-38]. A considerable portion of this variation can be assigned to deterministic factors such as direction and habitat characteristics [39,40], and/or explained by context-dependent mechanistic models incorporating landscape heterogeneity and vector movement characteristics [41,42]. Yet our methods for describing and modeling these patterns remain fairly limited, and theoretical studies have done little to elucidate their consequences.

In many systems, the probability of a seed ending up in a particular location depends on the type of substrate or habitat at that location – deposition is essentially habitat-specific [34]. A special case of this is when seeds are disproportionately deposited in habitats favorable for seed and seedling success; this “directed dispersal” has received considerable attention [39]. Yet this is just a small part of a larger phenomenon, with little attention to the opposite pattern of disproportionate dispersal to less favorable habitats. For example, several much-cited studies document directed dispersal into canopy gaps in a few neotropical forest taxa [43]. However, a community-level study found that overall seed arrival in gaps was much lower than seed arrival in the shaded understory for all functional groups [44]. Habitat-specific dispersal is common in both wind- and animal-dispersed seeds and pollen. For wind dispersal, habitat-specific deposition may result from the way in which seed or pollen movement is affected by topography and canopy structure [42,45-48], and/or by substrate characteristics determining the likelihood of secondary dispersal by wind [49]. For animal-mediated dispersal, habitat-specific deposition results from habitat preferences of seed dispersers or pollinators, both for movement in general [50,51], and for activities related to deposition (in the case of seeds), such as caching and defecating [52].

A challenge for modelling habitat-specific deposition is that deposition probabilities depend not only on fine-scale local habitat heterogeneities, but also on the habitat matrix of a larger area [53]. Thus increasing or decreasing propagule deposition probability by a fixed factor depending on habitat is too simplistic. Schurr *et al*. [54] address this challenge by first transforming physical space into “movement space”, reflecting areas of low and high permeability to (seed) flow by wind, and then evaluating dispersal kernels in this transformed space; this approach seems well-suited to modelling wind dispersal of both pollen and seeds. For animal dispersal, detailed spatially explicit models can simulate the influences of animal behavior and habitat structure on seed [41,50,55,56] and pollen [29,51] dispersal patterns. The parameterization and application of such models has become ever easier due to advances in animal tagging and telemetry, remote sensing (including accurate geo-referencing), and computation [43,57-60]. A key limitation is that such models are generally based on purely phenomenological descriptions of animal displacement kernels and habitat choice. Future research should aim to develop mechanistic descriptions of the processes behind such patterns, including internal motivation, memory, territoriality, and propagule retention (e.g. seed digestion, [61]) time models, thereby allowing for extrapolation to other spatial and temporal contexts [62]. These models could build on the extensive literature on animal movement ecology, which remains under-utilized to date by scholars working on plant dispersal [63-65].

Seed and pollen dispersal are also often anisotropic [66-71], whereas the standard distance-only models assume direction does not matter. Directional bias increases clustering and may thereby reduce the benefits of dispersal [37]. Importantly, anisotropic dispersal of pollen and seed will strongly influence mating patterns (e.g., correlated paternity), gene flow and spatial genetic structuring of neutral and adaptive genes. In the case of dispersal by animals, anisotropic patterns are generally related to the relative location of source trees, animal home ranges, and habitat, and can potentially be explained and reproduced by mechanistic models. In the case of dispersal by wind or water, anisotropy reflects the directionality of the dispersal vector, and/or asymmetries in the distribution of favorable deposition sites around the source. It is relatively straightforward to reproduce anisotropic patterns in mechanistic models of dispersal by wind or water, given data on the directionality of the dispersal vector [26]. However, most field studies of dispersal by wind simply integrate predictions and data over all directions [72]. This may in part reflect the challenge of describing anisotropic patterns with phenomenological models and the fact that anisotropic dispersal kernels invariably involve more parameters than isotropic ones and require larger samples to be fitted. Van Putten *et al*. [73] introduced a general framework for phenomenological anisotropic kernels that includes all previous such kernels (referenced in [73]) as special cases. Future research should better describe anisotropic dispersal patterns with available statistical tools, explain these patterns mechanistically, and evaluate their consequences for plant populations.

### 3. How can we measure long-distance dispersal across plant species’ ranges?

Long-distance dispersal (LDD) can be defined in absolute terms as the fraction of dispersal events that occurs above a given threshold distance associated with the biology, demography and environment of the species [4]. LDD of seed and pollen is important to the speed of colonization or invasion, metapopulation dynamics, long-distance gene flow, local adaptation, adaptive evolution [74], and demographic and genetic effects of fragmentation [75]. Island colonization and dispersal biogeography studies have demonstrated the potential for effective plant dispersal over scales of hundreds to thousands of km, and how understanding vector characteristics enable predictions about long-distance plant migration routes over extended time periods (e.g., [76,77]). Future studies on this front should build on more explicit mechanistic models of the interaction between vectors and propagule traits (see Section 1), and account for species’ establishment niches and potential arrival habitats, to provide a sounder hypothesis-testing framework concerning the source, path and effective establishment sites of long-distance propagules [78]. Dispersal biogeography approaches are limited in that they are difficult to apply within continents and cannot generally estimate dispersal rates [79]. Tallying the arrival of different gene lineages into islands may shed more light on the frequency and origin of LDD [80], but this approach only provides minimum frequency estimates, because immigrant lineages may have gone extinct through competition, drift or selection.

More general models are available to infer historical (i.e., averaged over generations) seed- and pollen-mediated gene dispersal rates among discrete populations using genetic structure data. These methods rely on simple demographic history assumptions to separate the genetic signature of dispersal from those of random drift and shared ancestral polymorphism [81,82]. Their spatial scale of analysis is potentially large, making them suitable for historical LDD inference, with the caveats that model misspecification and unsampled populations can bias dispersal estimates [83,84], and that current and past population distributions need not coincide, which complicates inferring the actual scale of dispersal estimates. In the case of continuously distributed species, theoretical studies have predicted how different LDD levels during range expansion should be reflected in contrasting genetic structures across newly colonized areas [85-90], but we still lack formal methods to use this kind of information. Future methodological advances will surely exploit the flexibility of Approximate Bayesian Computation (ABC) methods for LDD inference from genetic structure data under realistic demographic assumptions, both for discrete and continuous populations *e.g*. [91,92], as well as the information about gene flow contained in linkage-disequilibrium patterns across whole genomes [93]. Efforts to adapt these tools to disentangle the relative contribution of seed versus pollen dispersal to historical gene flow rates, either with uni- or biparentally inherited markers, would be a welcome addition for plant ecologists, as this topic has remained notably underexplored since the basic island model in [94] and [95].

Although historical LDD is interesting for biogeographic, population genetic and evolutionary studies, broad-scale patterns of dispersal under current (non-equilibrium) demographic and environmental conditions are becoming of greater concern. We anticipate growing emphasis on contemporary dispersal research spanning increasingly larger scales, using spatially and environmentally explicit approaches, and distinguishing effective dispersal (leading to successful establishment or reproduction) from basic dispersal (encompassing only propagule movement from source to deposition sites) [96]. Tracking recent or ongoing range expansions will remain a reliable source of information about the range of effective dispersal and the speed of migration into new habitats [97,98], while offering methodological advantages to establish recent LDD contributions to population establishment and growth [99]. A more general problem will be to estimate contemporary seed and pollen dispersal rates between discrete populations, or between localities throughout continuous plant ranges, accounting for or jointly inferring the effect of relevant spatial, demographic and environmental factors determining basic and effective dispersal. Mechanistic models provide a good basis for this purpose, but they are hard to validate over broad distances and do not easily reflect post-dispersal processes leading to effective dispersal [74,96]. Genetic methods are harder to extrapolate beyond the sampling area, but they can provide data at multiple spatial scales to validate mechanistic predictions, and estimate either basic or effective propagule dispersal with appropriate choice of sampling protocols and statistical analyses [96,100]. This flexibility of genetic methods can be exploited to investigate processes operating between the dispersed-seed and established-seedling (or between the dispersed-pollen and viable-embryo) stages, which increase spatiotemporal variation in effective dispersal patterns (see Sections 4, 5 and 7). Overall, scaling up mechanistic or genetic methods alone is unlikely to succeed for estimating contemporary seed and pollen dispersal rates (either basic or effective) over broad scales [74], so we suggest combining both.

The combination of mechanistic and genetic methods could be formalized within an ‘inverse problem’ framework: parameter estimates of the underlying mechanisms are retrieved from the (noisy) observation of resulting spatial patterns through mechanistic-statistical models [101] or state-space models [64], which associate a mechanistic model for the biological processes of interest to a statistical model for the observations. Inverse methods are increasingly popular for investigating large-scale biological mechanisms in general [102], and particle dispersion from unknown sources in particular [103], thanks to increasing computational power and the development of numerically intensive statistical methods (Bayesian MCMC, ABC). Concerning the statistical “component” of our problem of dispersal among discrete populations, genetic assignment methods (reviewed in [104]) are an appealing choice, because they overcome substantially the spatial scale limitation of genetic parentage analysis. Moreover, some developments of these methods explicitly estimate recent migration rates among populations [105-107], and specifically seed (or seedling) and pollen migration rates [108-110], defined as the proportion of propagules immigrating into a population. These procedures easily admit the incorporation of mechanistic formulations of seed and pollen migration rates (see Additional file 1), thus moving from the estimation of seed and pollen migration rates themselves to the estimation of the parameters of a mechanistic model for these rates [106,111].

For wind-dispersed propagules, mechanistic models for among-population migration rates could embed regional wind data in the form of connectivity maps, describing the probability of basic seed or pollen dispersal along possible trajectories linking a set of locations [77,112], as well as sub-models for propagule mortality during transport [72,113], mortality between seed deposition and seedling establishment for effective seed dispersal [114], and flowering phenological synchrony [115] and cross-population pollination rates [116] for effective pollen dispersal. Considering animal-driven seed or pollen dispersal in spatially heterogeneous landscapes, the mechanistic component for the connectivity network could build upon previous work on diffusive movement in patchy populations or metapopulations [64]. Some simple movement behavioral models indeed enable the analytical derivation of pairwise migration rates considering the structure of the entire landscape and not only the two populations considered (e.g. [117,118]), while new automated track annotation systems can help calibrate such behavioral models [60] (see also Section 2). Additional submodels would be necessary to include the pollen carry-over by individual pollinators or retention time of individual seed-dispersers, which may be particularly important for LDD events over continental scales [119]. In mosaic landscapes, the use of resistance surfaces to build connectivity maps (using least-cost distances, ecological distances or resistance distances) is also a promising approach [120]. This approach focuses extensively on the effect of land-use on dispersal, but methods are still needed to reliably parameterize the resistance values [120,121]. Finally, several types of observations resulting from the same processes could be analyzed simultaneously using mechanistic-statistical modeling, especially when associated with hierarchical Bayesian statistics [122]. Future studies should thus take this opportunity to estimate process parameters not only from genetic data but also from demographic, capture-recapture or presence-absence data [123,124]. The complexity of models including all these elements and the challenge of obtaining ecological data to parameterize them may be daunting, but we have reached the point where sufficient knowledge about the separate elements is available to attempt a multidisciplinary integration into useful inferential and predictive frameworks.

This combined genetic-mechanistic framework might also be applied to continuously distributed species, provided genetic assignment remained feasible. If significant clinal genetic variation were present over the spatial scale of the dispersal study, genotypic probabilities for dispersed propagules at any given location could be expressed as a continuous function of distance along the allele-frequency cline, potentially enabling the estimation of the LDD component along this direction. In the case of non-clinal (patchy) spatial genetic structure, allele frequency smoothing techniques may allow genetic assignment of propagules to a set of sampled and unsampled sources across the species range [125,126], although the accuracy of this method under contrasting sampling, dispersal, and genetic structure scenarios remains to be tested. If the number of migrant propagules is large (unfortunately an unlikely case for LDD), it may also be possible to use the genotypic composition of the propagule sample to help infer propagule migration rates from a known [127] or unknown [128] number of unsampled locations.

### 4. How variable are dispersal kernels among individuals and populations and what are the most important factors contributing to this variation?

Plant dispersal kernels are expected to be phenotype- and environment-dependent, given the number of intrinsic and extrinsic variables influencing the release, transport and settlement of seeds and pollen. Less evident is the relative weight of each variable, and how dispersal kernel variation is hierarchically distributed across individuals, populations and species, as well as over time. We deal with temporal dispersal variation in Section 5, and focus here on interindividual and interpopulation variation in dispersal kernels. Among-species variation in multivariate phenotypes putatively associated with dispersal (dispersal ‘syndromes’) can be substantial, and is usually interpreted in terms of vector specialization, resulting in potentially large differences in propagule dispersal kernels [7-9,129,130]. However, intraspecific variation has been shown to be as large as or even larger than interspecific variation for particular dispersal traits of some animal species, as a consequence of genetic variation among and within populations and of individual phenotypic plasticity [131]. Although analogous hierarchical quantitative analyses are still missing in plants, similar results could be anticipated, because substantially different seed and pollen dispersal estimates have been obtained among populations with contrasting density, parental architecture, and vector characteristics, both for wind- and animal-mediated dispersal [132-137]. Further comparative studies of propagule dispersal in multiple sites and populations would be advisable to overcome common methodological limitations in previous studies, such as unbalanced sampling designs, narrow spatial and temporal sampling scales, poor or absent uncertainty assessment of the difference in dispersal estimates (but see [138]), and insufficient or null replication across sites differing in intrinsic or extrinsic factors of interest. It will then be possible to move from the mere assessment of dispersal variation towards a hypothesis-driven identification of its environmental, demographic and phenotypic determinants. For this purpose, it would be advisable to combine empirical measurements of dispersal kernel parameters with mechanistic predictions based on measurements of vector occurrence and characteristics, environmental variables, and plant dispersal traits, along the principles suggested in Section 1.

At a narrower spatial scale, dispersal kernel variation within populations is primarily caused by local-scale heterogeneity in phenotypic dispersal traits and/or by the effects of local environmental variation on dispersal vectors (e.g., wind and frugivore behavior, Section 2) [42,54,56,139,140]. Changes in dispersal distances should also be expected among individuals with different pollen shedding or seed release phenology, if the different vector contributions and/or behaviors vary throughout the season [141,142]. In addition, differences in microhabitat, age, and genotype may produce variation in parental (e.g. plant height) and propagule (e.g. fruit or seed size) phenotypic traits associated with dispersal [143-145]. However, dispersal kernels are generally considered constant within populations, probably because this is assumed by statistical approaches typically used to fit observed patterns of dispersal [15,18,19,35]. Future models could attempt to estimate the within-population distribution of dispersal kernel parameters and their association with local phenotypic and environmental variables, using either mechanistic approaches [41] or extensions of recently developed genetic methods to estimate individual variation in dispersal parameters [146,147]. The latter methods could also estimate the association between dispersal kernel parameters and reproductive success, which, to the extent allowed by sampling and spatial scale limitations, would start shedding light on the individual fitness consequences of short- and long-distance dispersal in particular environments. Estimates of individual variation in dispersal kernel parameters could also be combined with quantitative genetics methods to estimate heritability in the wild [148], as a first step to evaluate the genetic determinism of dispersal traits.

Gathering empirical information about intraspecific variation in seed and pollen dispersal kernels and its phenotypic and environmental drivers will contribute to the construction of more realistic models of species distribution and interactions in changing environments (see Sections 6, 7 and 8), while determining what proportion of this variation is genetically determined will be essential for assessing the potential for evolution of dispersal in future environments [149]. There are a few well-documented cases of rapid seed dispersal evolution during colonization [150-154] and after habitat fragmentation [155,156], but these evolutionary responses will probably be highly variable across taxa, owing to differences in standing genetic variation, trait heritability, phenotypic plasticity and fitness effects of dispersal traits [157]. Important insights could be obtained from phenotyping individual dispersal traits and dispersal kernels in common garden experiments replicated in contrasting environments [158]. In conducting these experiments, it would be ideal to (i) measure the short- and long-distance components of seed and pollen dispersal kernels and their presumed phenotypic and environmental correlates; and (ii) assess potential correlations between dispersal and other phenotypic traits of ecological relevance, which might represent multivariate genetic constraints on dispersal evolution [159].

### 5. How temporally variable is dispersal and what are the implications of this variation for plant populations and communities?

Dispersal varies not only over spatial scales (see Sections 2 and 4), but also over time scales, from seconds to weeks to years, due to temporal variation in endogenous and exogenous factors influencing dispersal. Wind speed and direction, including wind turbulence, vary temporally due to both variable atmospheric forcing and varying local leaf area density, vegetation structure, and landscape configuration [10,141]. Pollinator and frugivore guild composition, abundance, and behavior also vary temporally [142,160,161], with behavior influenced especially by the local abundance and spatial distribution of other flowering and fruiting plants [162,163]. There is also temporal variation in the physical condition and form of the diaspore and of tissues involved in seed release or abscission in wind-dispersed species [164], or in plant traits that attract and reward animal dispersers [165,166].

Most dispersal studies disregard this temporal variability, yet it critically affects the interpretation of dispersal data. Because of temporal variation, sampling duration and timing can strongly affect dispersal estimates [167]. The standard approach is to implicitly average over temporal variability, providing time-integrated measures of dispersal over the season or seasons of study (e.g. [168]). The few studies that have evaluated dispersal in multiple seasons or years have found significant temporal variation, for both pollen [169] and seeds [36,170]. This calls into question our ability to draw conclusions about dispersal in systems in which data collection spans only one or a few seasons or years, as is the case in the vast majority of empirical dispersal studies.

Temporal variation in dispersal has important implications for plant populations. Inter-annual variation in pollen and seed dispersal can determine mating system variation [169], the assemblage of genetic diversity during regeneration [171] and the heritability and the response to selection of dispersal-related traits [149]. This is especially relevant for long-lived species, where the contribution of individuals to population demography and genetics spans over multiple reproductive and dispersal episodes [172]. Knowing the extent of temporal variation in dispersal could also shed more light on the consequences of masting for population dynamics, because masting benefits could be influenced by temporal covariation between seed crop size and spatial patterns of seed dispersal, a potential association that remains largely unexplored (but see [173]). More generally, establishing temporal covariation patterns between environmental variables, reproductive rates, seed and pollen dispersal patterns, and effective seedling establishment rates will shed light on the frequency of the rare favorable years on which successful recruitment of long-lived species may disproportionately depend [174], and their effect on the evolution of pollination and dispersal strategies, the speed of population migration under climate change (see Section 7) and the spread of invasive species [175,176].

We thus advocate and expect more studies measuring temporal variation in seed and pollen dispersal, its mechanistic determinants, and its consequences for populations and communities, much as we have advocated for studies of spatial variation in dispersal (Section 4). Temporal characterizations of seed and pollen dispersal should go beyond measuring variation in fecundity to examine fluctuations in dispersal distances and landscape-dependent dispersal patterns (Section 2), and their association with focal plant conditions and vector dynamics. The task can be enormous when dealing with complex ecological networks or large landscapes; a comprehensive understanding of temporal variation in dispersal will probably require conceptual and methodological advances to establish a clear partition of dispersal variability into environmental, spatial and temporal components (see [169] for a comparable scheme applied to mating systems) over different nested scales.

Insofar as temporal variation in seed dispersal is an important contributor to temporal variation in recruitment success, it also becomes a critical component of studies of community dynamics, and specifically the potential for species coexistence via temporal niche differentiation, also known as the “storage effect” [177]. In this context, a critical question concerns the degree to which temporal variation in seed dispersal is synchronous or asynchronous among species. Temporal fluctuations in wind speed or frugivore abundance might be expected to lead to synchronous variation, while competition for shared frugivores could lead to asynchronous variation [161]. Studies are needed to evaluate the consequences and importance of temporal dispersal variation at multiple scales for plant communities. In particular, long-term multi-species studies should investigate how coexisting species co-vary in their temporal patterns of seed dispersal, and quantify associated contributions to interspecific patterns of temporal variation in recruitment. To address these multi-species questions, much is expected from advances in the spatial analysis of plant-plant and plants-frugivore networks [140] that incorporate demographic and genetic aspects of focal species or populations [178,179].

### 6. What is the actual importance of seed dispersal in determining community processes and patterns?

Seed dispersal is one of four fundamental processes in community ecology, the others being selection (deterministic differences in per capita growth rates among species), drift (stochastic changes in species abundances), and speciation [180]. The importance of seed dispersal for community patterns of species diversity, abundance, and composition is generally accepted; indeed, it is often stated in introductions and discussions of empirical studies of dispersal. Further, theoretical studies clearly show that seed dispersal or migration rates strongly influence community patterns in neutral and niche models (e.g., [181,182]). However, there is a scarcity of empirical studies convincingly demonstrating the role of seed dispersal rates and patterns for community dynamics and structure [183].

Several types of empirical studies to date have provided insights into the role of seed dispersal in community patterns, but each has major shortcomings. Empirical analyses of species turnover in space (beta diversity) often invoke seed dispersal as the explanation for distance-dependent patterns not explained by environmental variation (e.g., [184]); however, these studies are inherently limited in their ability to distinguish the influence of dispersal from that of environmental niches [185], do not consider distance-independent variation in dispersal, and generally include no link to empirically measured dispersal (but see [186]). Empirical studies of variation in community patterns with differences in isolation/connectivity and hence presumed seed dispersal/migration rates generally find strong relationships, but these studies usually have important confounding factors – e.g., differences in the abundance and species composition of animals that interact with plants [187] or in the quality of habitat patches [188]. Studies comparing areas with and without vertebrate seed dispersers, whether due to differences in hunting pressure or to experimental exclusion, are similarly confounded by variation in vertebrate seed predation and herbivory [189,190]. Numerous experimental seed addition studies have shown that species diversity and composition often responds strongly to seed availability – but these studies effectively simulate alterations in fecundity as much as or more than they do dispersal [191].

The lack of good empirical tests of theory on the importance of seed dispersal to communities in part reflects a mismatch between the simplistic way in which dispersal is generally represented in models and the more complex dispersal patterns observed in most real ecosystems. Most models set seed dispersal rates as identical for all species, when in the real world dispersal rates invariably vary greatly among species within communities. Further, a common approach is to model seed dispersal as a dichotomy of within-patch vs. between-patch dispersal using a metacommunity framework [192,193]. Few real-world ecosystems are well-approximated by such models, especially when all patches are assumed equally connected, as is generally the case. The alternative is spatially explicit models of communities. Advances in computing and in mathematical techniques, particularly moment methods, have made these models increasingly accessible and tractable, and has led to a tremendous increase in relevant theoretical work [194-197]. This work has expanded our understanding of how seed dispersal can affect community patterns in theory, both alone and in interaction with selection and drift.

We believe that two alternative approaches offer the best potential to advance our understanding of the role of seed dispersal in community patterns – not only spatial patterns of turnover, but also relative abundances, species composition, and diversity. The first is large-scale field experiments manipulating dispersal patterns. Such manipulations should involve not only seed addition, but seed redistribution within areas of study. These might for example involve extending or restricting seed dispersal for all species, and/or homogenizing seed dispersal patterns across species. Few seed redistribution studies have been undertaken even for individual species at small scales [198], much less communities. The second approach is to adroitly combine empirical and theoretical work, by collecting empirical data on seed dispersal and competitive interactions for multiple species sufficient to parameterize simulation models that reproduce relevant community patterns, and then using these models to conduct simulation experiments regarding the effects of altered seed dispersal on community patterns. For example, Ribbens *et al*. [15] and Pacala *et al*. [199] take such an approach to examine the importance of seed dispersal to a temperate forest community, parameterizing the spatially explicit, individual-based model SORTIE, and then evaluating the sensitivity of species relative abundance and other community patterns to changes in dispersal parameters. More studies of these kinds are needed to establish how seed dispersal matters not only to populations, but to communities.

### 7. How will dispersal influence population viability under climate change?

We now take a long-term perspective to examine the importance of seed and pollen dispersal in the complex interaction between demographic dynamics and adaptive processes in a changing climate. A more conventional title for this section could have run “*Will plants migrate fast enough to avoid extinction under climate change?*”, but this potentially misleading question suggests that tracking suitable habitats through migration is the only mechanism by which plants can avoid extinction in a dynamic environment, disregarding genetic adaptation and adaptive phenotypic plasticity. Paleoecological records, especially rich for woody plants, suggest that latitudinal and altitudinal displacements from multiple refugial sources have been the main responses of many plant species to past climate changes [200-202], but migration probably has interacted and will interact with genetic evolution, gene flow and phenotypic plasticity. For instance, climatic tolerance and dispersal capacity can both evolve during migration [74,203,204], effective pollen or seed dispersal among distant populations may favor adaptation to new conditions [74,205], and adaptive phenotypic plasticity may buy time for migration, as it buffers the demographic effects of maladaptation [206]. A more relevant question would thus be whether the joint action of dispersal, genetic evolution and phenotypic plasticity will be sufficient to avoid the extinction of particular populations under the novel selective pressures brought by climate change, given population-specific factors such as census and effective size, current climatic tolerance, interspecific interactions [207], geographic range position [208], landscape connectivity [209], gene immigration from other populations [74], levels of standing multivariate genetic variance [210], and multivariate genetic constraints to adaptive evolution [159,211]. Even if we will probably see the consequences of climate change before being able to answer such a question, it remains relevant to rank populations according to estimated extinction risk, and to identify the main natural and anthropogenic factors reducing their viability, including dispersal limitation.

Efforts to incorporate this complexity in the prediction of climate-driven species range shifts are heading to the combination of simple habitat models with mechanistic spatially-explicit models of metapopulation dynamics [212-215], genetic and phenotypic adaptation [208,216,217], and species interactions [218,219]. Future work should deal with knowledge gaps that are critical for linking the different components of these models, such as the effects that long-distance seed and pollen dispersal (along with genetic adaptation and phenotypic plasticity) have on population fitness and demographic dynamics, as well as the potential feedbacks between demographic, ecological and evolutionary processes [206,207,220-223]. Plant movement ecologists can make important contributions to this multidisciplinary endeavor by formulating and fitting realistic individual and gene movement modules that are interactive with the ecological and demographic layers of range-shift models. Rather than assuming invariant migration, population spread models should use mechanistic descriptions of seed fecundity, transport and establishment, enabling the integration of relevant phenotypic, climatic, and ecological factors that determine variation in the seed dispersal kernel (see Sections 1, 2, 4 and 5). Recent works have weighed the relative effects of some of these factors on plant population spread, including seed and maternal plant morphology, wind conditions, non-random seed abscission, animal movement and seed retention time, seed fecundity, plant maturation age, plant longevity, and environment-dependent post-dispersal mortality [37,224-229]. From these studies, it is becoming clear that post-dispersal factors determining effective establishment and growth are as important or more than long-distance seed transport in determining the speed of plant migration. We therefore need not only better LDD data, but also further studies to characterize niche variation across plant life stages, from seed germination, through seedling establishment, to adult survival and reproduction [230-233]. We will then be in a better position to understand how the interplay between LDD, niche requirements, and dynamic heterogeneous environments (including fragmented habitats with variable abundance of mates, dispersers, predators and competitors) determines the speed of spread of plant populations under climate change.

Species distribution models allowing for genetic evolution should also include realistic modelling of seed- and pollen-mediated gene flow among populations across shifting ranges, since both are expected to influence local adaptation and niche evolution [74,223]. In Section 2 we outlined a mechanistic framework for modelling seed and pollen migration rates among populations that would be amenable to integration into future broad-scale species distribution models, because it can account for spatial, demographic, and environmental determinants of long-distance propagule transport probabilities, and can be fitted empirically using genetic marker information. It would thus be possible to obtain a measure of the regional ecological neighborhood to which a focal population is exposed through gene immigration (similarly to [234], but weighted by contemporary propagule transport probabilities). The ecological and genetic layers of the model could then determine the probability of establishment of seed immigrants or *hybrids* and their potential population fitness consequences, conditioned on the habitats of origin and arrival and the species niche across life-stages. Ultimately, any quantitative prediction about population viability will be sensitive not only to model selection but also to the choice of parameter values. Future transplant and controlled-pollination experiments should help us quantify the probability of effective establishment for long-distance seed and pollen migrants under varied biotic and abiotic environments [235].

### 8. Will dispersal across anthropogenic landscapes in a globalized world be limited or enhanced?

Human activities have become a key driver of plant dispersal, both through their direct contribution to the transport of propagules (e.g., [236,237]) and through anthropogenic changes in land use, habitat fragmentation, biotic connectivity (resulting in biological invasions) and climate change [238,239]. All these factors represent important global drivers of genetic erosion, species extinction and biodiversity loss [240,241]; hence, understanding their combined impact on seed and pollen dispersal represents a challenging but tremendously important task. In turn, increasing our current understanding of plant dispersal has been identified as a critical factor to obtaining reliable prediction of plant responses to global environmental change (GEC hereafter) (see Section 7 and [242,243]).

The prospects are particularly worrying for animal-mediated pollen and seed dispersal, because plant-animal mutualisms tend to be negatively affected by most drivers of GEC [244]. For example, habitat fragmentation, biological invasions and climate change negatively affect outcross pollination and mating patterns of insect-pollinated species (reviewed in [245] and [246]). Cascading effects of reduced pollination on seed dispersal by animals could be exacerbated by direct effects of climate on fruiting phenology [247], the disruption of seed-dispersal mutualisms by invasive species [248] and impaired dispersal among habitat fragments [249]. Effective seed dispersal may be reduced further by associated increases in seed and seedling predation (e.g., [250]). These effects vary among plant species, depending in part on their morphological or functional traits. For example, large-seeded species tend to show stronger reductions in seed dispersal and stronger decreases in seed predation as a result of fragmentation (e.g., [251,252]), largely owing to the defaunation of smaller fragments (i.e., the selective removal of large-bodied dispersers and predators; e.g. [253,254]).

Generalizing the effects of multiple drivers of GEC on communities is challenging, because communities are interlinked by interactions of variable sign and strength, and because these effects are likely to be scale- and species-dependent (e.g., [255] for the response of pollinators to land use changes). This task will probably require a “patchwork” of approaches, including (i) correlational landscape-level approaches to infer relationships between drivers and response variables and determine how they scale over space and time; (ii) comparative studies that identify adequate predictors of species’ responses to GEC based on morphological, behavioral and functional traits, and estimate their effects on species interactions and interaction networks; (iii) mechanistic studies based on detailed information of representative systems, in which seed dispersal models based on individual, rule-based descriptions of animal movement are used to generate scenarios of broader-scale responses to GEC (see Sections 2 and 7); and (iv) experimental manipulations of fragmented and/or anthropogenic landscapes (e.g., patch characteristics, habitat corridors or landscape features influencing matrix permeability [256-259]) to test predictions regarding planned landscape modifications undertaken for management purposes (using, whenever possible, an adaptive approach; [260,261]). The combination of these four approaches could provide more accurate estimates of the responses to anthropogenic pressures acting on different species assemblages, for various spatial arrangements, management regimes and temporal scales.

On first principles, the effects of GEC on wind-mediated pollen and seed dispersal should be more straightforward [262]. Empirical results and theoretical predictions suggest however that this is not necessarily the case, because some fragmented plant populations exhibit enhanced wind dispersal of seed and pollen while others show the reverse trend [75], and because different assumptions about future wind speeds lead to opposite airborne propagule dispersal predictions [225,228,263]. It is clear that variation in wind-mediated dispersal mechanisms should determine interspecific differences in dispersal sensitivity to habitat alteration and climate change, but some of the conflicting results in the literature seem to be the consequence of (i) a poor characterization of habitat and demographic disturbance over relevant spatial scales, relative to seed or pollen dispersal range, and (ii) high uncertainty about future local and regional wind regimes, two issues that deserve more careful attention. Efforts to predict the effects of GEC on wind dispersal are especially hampered by the difficulty of modeling LDD (see Section 3) and its interaction with the spatiotemporal variability that often characterizes anthropogenic landscapes (e.g., [264]). Long-distance wind dispersal of plant propagules depends on phenomena, such as turbulent updrafts and downdrafts, that vary strongly with local and regional weather conditions, micro-topography, foliage density, and canopy and habitat structure [3,10,11,46,265]. Predictions are certainly aided by the increasing refinement of mechanistic models [40,72,225,227,265,266], but these need to be better validated if low frequency events are to be reliably predicted. The integration of genetic and mechanistic models bears the strongest potential for this task (Section 3). On the other hand, extrapolation of model predictions across species or functional types can be used to derive approximate generalizations about vegetation responses to GEC (e.g., [7-9,130]), which should be tested using correlative studies and experimental manipulations in real landscapes (as advocated above for animal-mediated dispersal).

Applications of this knowledge to the management of anthropogenic landscapes must factor in the potential consequences of pollen and seed dispersal across such altered landscapes – consequences that may be positive or negative overall [267,268]. Gene flow tends to increase genetic variation within populations, limiting inbreeding depression and increasing evolutionary potential, but it may also limit local adaptation owing to introgression of maladapted genes and the disruption of co-adapted gene complexes [269-272]. A comparable duality of effect may be expected at the community level, with increased connectivity enhancing local population persistence and alpha diversity – but tending also to increase homogenization (reducing beta diversity) and facilitate the arrival of invasive species, pathogens and parasites [273-276]. This is particularly important in current scenarios of rapid climate change, in which habitat fragmentation and the establishment of foreign genotypes and species may constrain the processes of local adaptation and geographic redistribution required for species and community persistence [268]. The evolving metacommunity framework provides a sound theoretical ground for advancing estimates on the optimal levels of connectivity in anthropogenic landscapes subjected to GEC (e.g., [277]), which could be validated and refined using management actions aimed at enhancing connectivity.

## Conclusions

Advances in plant dispersal ecology research will be determined by our ability to surmount challenges of spatial scale and heterogeneity, temporal scale, and system complexity. Enlarging the spatial scale of empirical studies will remain a necessity to avoid biased descriptions of dispersal and its ecological and evolutionary consequences. New inferential and predictive schemes should be developed and applied to better describe the rate and trajectories of effective seed and pollen migrants over different spatial scales in environmentally and demographically explicit context, incorporating landscape-dependent components of vector and propagule movements. This will probably require a combination of mechanistic and phenomenological (e.g., genetic-based) approaches that, in the unavoidable trade-off between spatial scope, sampling intensity and accuracy, should seriously assess expected statistical power and uncertainty for low-frequency (but still ecologically and evolutionarily important) dispersal rates, model miss-specifications, and limited sampling. Temporal scale issues will pervade plant dispersal ecology studies, from a more meaningful characterization of average dispersal patterns given variation in dispersal within and among seasons, through the assessment of the consequences of such temporal dispersal variation for population and community dynamics, to long-term predictions about population and species persistence based on observed and modelled feedbacks between dispersal, demography and evolution in changing environments. Finally, sufficiently approximating the dynamic complexity of environments, ecological networks and communities will be essential for characterizing all relevant biotic and abiotic mechanisms driving plant dispersal and their sensitivity to global change, and for better understanding the ecological consequences of dispersal in changing environments. We will certainly need to increasingly pool data and expertise from multiple disciplines to meet these big challenges, for which we advocate not only further cooperative research efforts, but also the implementation, standardization and usage of open repositories of dispersal data and models.

## Additional file

Additional file 1:
**Combining genetic and mechanistic approaches for long-distance dispersal estimation.**

## Abbreviations

ABC: Approximate Bayesian computation
GEC: Global environmental change
LDD: Long-distance dispersal
