# Supplementary material for: Space, time and complexity in plant dispersal ecology
Source: Mov Ecol. 2014 Aug 1;2:16. doi: 10.1186/s40462-014-0016-3 (PMC4337469; doi:10.1186/s40462-014-0016-3)
Supplement: Additional file 1: — Combining genetic and mechanistic approaches for long-distance dispersal estimation. [file 40462_2014_16_MOESM1_ESM.docx]

**Combining genetic and mechanistic approaches for long-distance dispersal estimation.**

Assume a set of discrete plant populations and denote *m_S,ij_* and *m_P,ij_* the seed and pollen contemporary migration rates among population pairs (*i*,*j*). Methods based solely on genotypic information (e.g. [1]) rely on the likelihood of observed genotypes in all populations *i* (and *j*) at times *t* and *T*, given all pairwise migration parameters:

$P\left(\mathbf{Geno}_{\mathbf{t}},\mathbf{Geno}_{\mathbf{T}}\left|m_{S,ij},m_{P,ij}\right.\right)$.

If some information is available about the location (*x*, *y*), phenology (*ph*), area (*A*) and local environment (*E*) of the source and recipient populations *i* and *j*, it would then be possible to model *m_S,ij_* and *m_P,ij_* as mechanistic functions

where *θ* are parameters of interest of the mechanistic model, to be estimated, and where ε includes the residual variability. Considering the hierarchy in the parameters, the mechanistic parameters *θ* can then be estimated using the likelihood of observed genotypes

.

Maximum-likelihood approaches can be implemented if computationally tractable, but, alternatively, the hierarchical nature of the model can be described using Bayesian methods [2].

**References**

1. Robledo-Arnuncio JJ: **Joint estimation of contemporary seed and pollen dispersal rates among plant populations**. *Mol Ecol Resour* 2012, **12**:299–311.

2. Clark JS, Gelfand AE: **A future for models and data in environmental science**. *Trends Ecol Evol* 2006, **21**:375–380.
